# Comparative transcriptome sequencing and *de novo* analysis of *Vaccinium corymbosum* during fruit and color development

**DOI:** 10.1186/s12870-016-0866-5

**Published:** 2016-10-12

**Authors:** Lingli Li, Hehua Zhang, Zhongshuai Liu, Xiaoyue Cui, Tong Zhang, Yanfang Li, Lingyun Zhang

**Affiliations:** 1 Key Laboratory of Forest Silviculture and Conservation of the Ministry of Education, Beijing Forestry University, Beijing, 100083 P. R. China; 2 Present address: College of Forestry, Northwest A&F University, Yangling, Shanxi 712100 P. R. China

**Keywords:** *Vaccinium corymbosum*, Transcriptome, Fruit development, Color development, Anthocyanin biosynthesis

## Abstract

**Background:**

Blueberry is an economically important fruit crop in Ericaceae family. The substantial quantities of flavonoids in blueberry have been implicated in a broad range of health benefits. However, the information regarding fruit development and flavonoid metabolites based on the transcriptome level is still limited. In the present study, the transcriptome and gene expression profiling over berry development, especially during color development were initiated.

**Results:**

A total of approximately 13.67 Gbp of data were obtained and assembled into 186,962 transcripts and 80,836 unigenes from three stages of blueberry fruit and color development. A large number of simple sequence repeats (SSRs) and candidate genes, which are potentially involved in plant development, metabolic and hormone pathways, were identified. A total of 6429 sequences containing 8796 SSRs were characterized from 15,457 unigenes and 1763 unigenes contained more than one SSR. The expression profiles of key genes involved in anthocyanin biosynthesis were also studied. In addition, a comparison between our dataset and other published results was carried out.

**Conclusions:**

Our high quality reads produced in this study are an important advancement and provide a new resource for the interpretation of high-throughput data for blueberry species whether regarding sequencing data depth or species extension. The use of this transcriptome data will serve as a valuable public information database for the studies of blueberry genome and would greatly boost the research of fruit and color development, flavonoid metabolisms and regulation and breeding of more healthful blueberries.

**Electronic supplementary material:**

The online version of this article (doi:10.1186/s12870-016-0866-5) contains supplementary material, which is available to authorized users.

## Background

Blueberry (*Vaccinium corymbosum*) is an economically important small fruit crop and a member of Ericaceae family which includes many species, such as blueberry, cranberry (*V. macrocarpon*), lingonberry (*V. vitis-idaea*), rhododendron, and more than 400 other species [1, 2]. Three major types of blueberry are harvested commercially including lowbush (*Vaccinium. angustifolium*), highbush (*V. corymbosum*), and rabbiteye bluberry (*V. ashei* or *V. virgatum*). Although mostly originated in North America, many blueberry species are widely grown in Asia, Europe, South America, Africa, Australia and New Zealand owing in part to their high level of vitro antioxidant capacities [3, 4]. Blueberry is becoming a major crop in China, cultivated widely from temperate area to subtropical region. There are currently three major areas for blueberry cultivation in China, the Jilin and Liaoning provinces, the Shandong provinces and the areas of the Yangtze River [5].

Demand and consumption worldwide of blueberry has greatly increased in recent years for its beneficial influence on human health. These positive effects are generally due to the high levels of flavonoid [6], which have been linked to improve night vision, prevent macular degeneration, and decrease the heart disease [7, 8]. Therefore, it is crucial to elucidate the molecular mechanisms that trigger biosynthesis and accumulation of anthocyanin metabolites during fruit and color development. The blueberry genome is large (600 Mb/haploid genome) and genomic information is limited compared to some plants like grape, for example [9], which restrains the dissection of blueberry. Over the past decades, more attention has been focused on the analysis of plant cold resistance, cultivation, and effects on human health [10, 11]. Since massive amounts of information can be obtained from genome-scale expression data, RNA sequencing has become a powerful technology to profile the transcriptome [12]. To date, RNA sequencing has been reported in bilberry (*Vaccinium myrtillus*) and cranberry [13, 14]. Recently, transcriptome sequences of blueberry were analyzed during cold acclimation and at different development stages of fruit by ESTs sequencing or RNA sequencing [2, 15]. So far, transcriptome sequences have been generated using next generation sequencing so far from northern highbush [2], half-high [16], and southern highbush blueberry [17]. However, the information is still limited regarding the control of horticultural traits such as the molecular regulation mechanisms of blueberry maturation and flavonoid metabolism.

In order to gain new insights into molecular mechanism at transcriptome level, we performed transcriptome sequencing and gene expression profiling for the northern highbush blueberry variety ‘Sierra’ over berry development with Illumina sequencing technique. A total of more than 13.67 Gbp of data were generated and assembled into 186,962 transcripts and 80,836 unigenes. Large numbers of simple sequence repeats (SSRs) and candidate genes, which are potentially involved in plant growth, metabolic and hormone pathways, were identified. In addition, RNA-Seq expression profiles and functional annotations have been made publicly available (accession number: SRR2910056). We believe that this study provides a new and more powerful resource for interpretation of high-throughput gene expression data for blueberry species.

## Results and discussion

### Sequence analysis and *de novo* assembly

The high quality Illumina sequencing data produced from *Vaccinium corymbosum* ‘Sierra’ during fruit and color development has been deposited in NCBI SRA database under accession number: SRR2910056. Three independent cDNA libraries were constructed from RNA samples of green fruit (G, 30d), pink fruit (P, 50d) and blue fruit (B, 75d), respectively. In total, after removing adaptor sequences, ambiguous nucleotides and low-quality sequences, 67,689,734 independent reads (13.67 Gbp) with more than 92 % Q30 bases were selected using Illumina HiSeq™ 2000 from the three samples (Table 1). The GC content of the three samples was approximately 46 %.

**Table 1:**
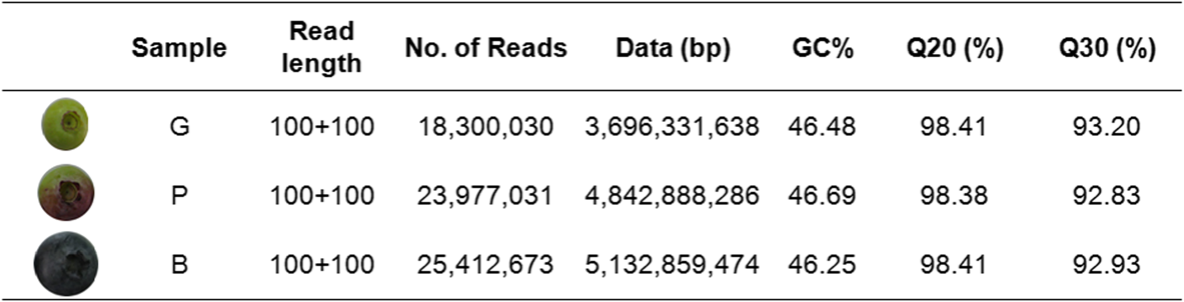
Sequencing the blueberry transcriptome at different stages of fruit development

Fruits at developmental stages of green, pink, and blue were sampled for transcriptome sequencing

Using the Trinity de novo assembly program [18], we assembled the short read sequences from the three samples into 186,962 transcripts. An overview of the assembled transcripts and unigenes are exhibited in Tables 2 and 3. The N50 value of the three sample assemblies was 1688 bp, and 66, 855 (36.95 %) transcripts were longer than 1 kb. The transcripts were subjected to cluster and assembly analyses, resulting in 80,836 unigenes (N50 value = 1204 bp), among which 15,457 (19.13 %) genes were greater than 1 kb. This is about 10 times greater than that in a previous report where a 17,134 ESTs resulted in a total of 8500 unigenes from two samples by Sanger sequencing [15]. Li et al. reported 57,331 unigenes in half-high blueberry from their two fruit samples (pulp and skin) [16]. Gupta et al. produced around 60,000 gene models from five stages of berry fruit development and ripening [17]. Therefore, we believe that the 13.67 Gbp of data generated and 186,962 transcripts and 80,836 unigenes assembled in this study are an important advancement and new resource for interpretation of high-throughput data for blueberry species whether regarding sequencing data depth or species extension. It is noteworthy that we have compared our dataset with the previously published Illumina dataset [17]. Compared with the previously published dataset, our dataset has a higher amount of data (Nucleotide), better data quality (Q20% or Q30%), and better assembly results (Average length and N50) (Additional file 1: File S1). There is no doubt that we now have a more powerful information database with higher capacity for gene expression analysis in *Vaccinium*, especially for fruit development and maturation, flavonoid metabolism and regulation and breeding for more healthful blueberries.

**Table 2:** The transcripts of blueberry

Transcripts of length	Total number	Percentage
200–300	39,000	21.55 %
300–500	35,561	19.65 %
500–1000	39,546	21.85 %
1000–2000	41,360	22.86 %
2000+	25,495	14.09 %
Total number	180,962	
Total length	186,855,896	
N50 length	1688	
Mean length	1032.57

**Table 3:** The unigenes of blueberry

Unigenes length	Total number	Percentage
200–300	29,377	36.34 %
500–1000	14,140	17.49 %
1000–2000	9665	11.96 %
2000+	5792	7.17 %
Total number	80,836	
Total length	56,528,444	
N50 length	1204	
Mean length	699.2978871	

### Similarity analysis

**Fig. 1:**
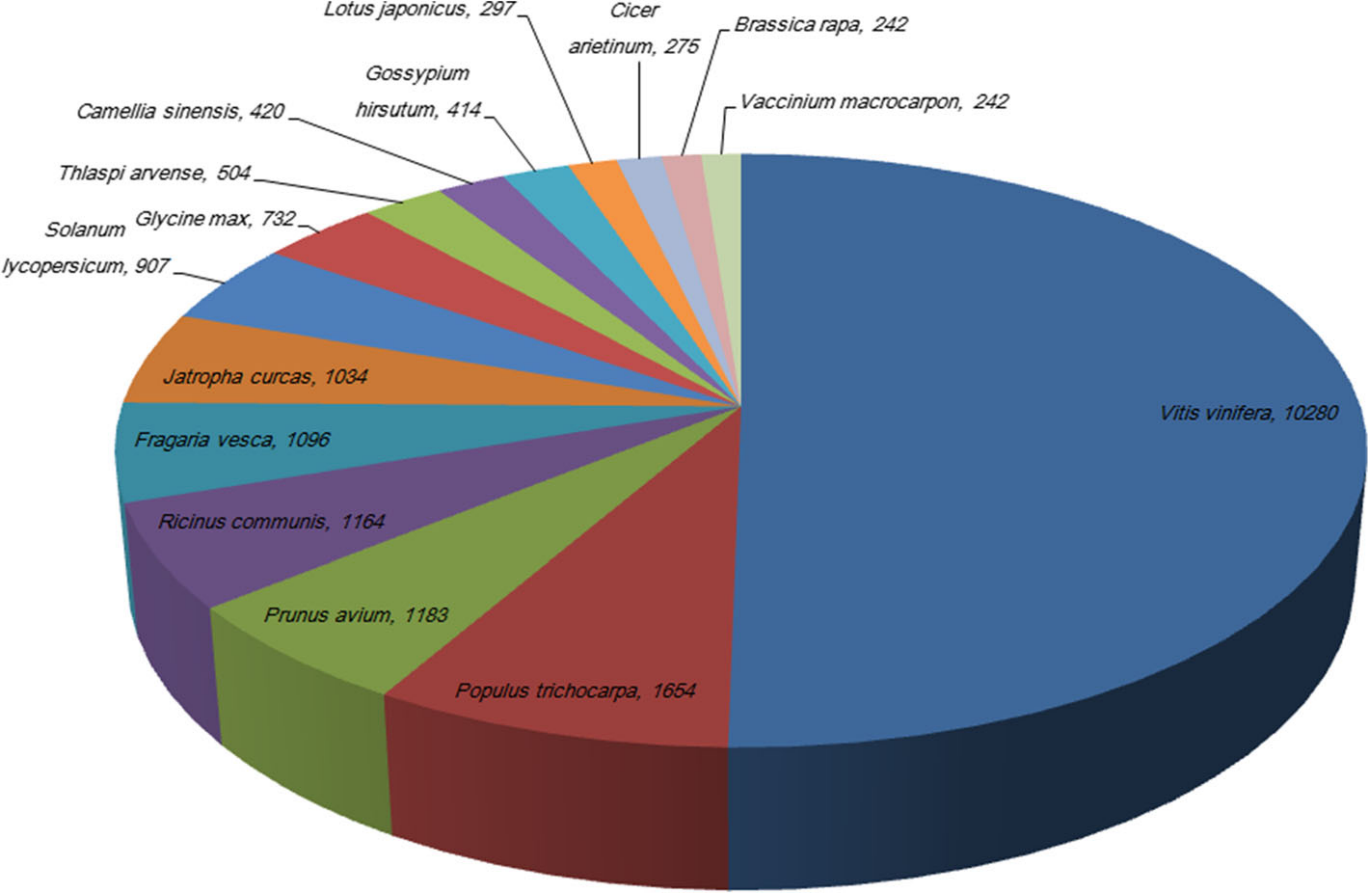
Species distribution of the top BLAST hits in Nr database. Values were number of unigenes in *Vaccinium corymbosum* homologous with those in other species such as *Vitis vinifera*, *Populus trichocarpa*, *Prunus avium*, *Ricinus communis*, *Fragaria vesca*, *Jatropha curcas*, *Solanum lycopersicum*, *Solanum lycopersicum*, *Camellia sinensis*, *Gossypium hirsutum*, *Brassica rapa*, *Vaccinium macrocarpon*

In order to better evaluate the similarity between blueberry and other organisms, blueberry unigenes were submitted to the non-redundant (Nr) NCBI database for BLASTx similarity analysis. The results showed that *Vaccinium corymbosum* unigenes hit a wide range of plant species including *Vitis vinifera*, *Populus trichocarpa*, *Prunus avium*, *Ricinus communis*, *Fragaria vesca*, *Jatropha curcas*, *Solanum lycopersicum*, *Solanum lycopersicum*, *Camellia sinensis*, *Gossypium hirsutum*, *Brassica rapa*, *Vaccinium macrocarpon*, etc. (Fig. 1). Among them, interestingly, 10,280 (12.72 %) unigenes showed a significant homology with sequences of *Vitis vinifera*, and 1654 (2.05 %) and 1183 (1.46 %) unigene sequences had a high similarity with those of *Populus trichocarpa* and *Prunus avium,* respectively. The high similarity of unigenes between *Vaccinium corymbosum* and *Vitis vinifera* suggests the possibility of using *Vitis vinifera* transcriptomes and genomes as a reference sequence for *Vaccinium corymbosum*. In contrast, only 242 (0.29 %) unigenes had a high similarity with sequences of *Vaccinium macrocarpon*, probably owing to the insufficient number of sequences in Genebank.

### Sequence annotation

To annotate the assembled sequences, several complementary approaches were utilized. The unigenes were annotated by aligning with the deposited ones in diverse protein databases (Nr, Nt, Swiss-Prot, KEGG, COG, GO and TrEMBL) and the best one was selected from the matches with an E-value of less than 10^−5^. The overall functional annotation is described in Table 4. Among the 80,836 unigenes, 10,263 (12.70 %) and 21,870 (27.05 %) unigenes had significant matches in the COG and GO database, 26,902 (33.28 %) in the Nt database, and 21,447 (26.53 %) in the Swiss-Prot database. The 34,006 (42.07 %) unigenes were annotated in the Nr, Nt, Swiss-Prot, KEGG, COG, GO and TrEMBL databases.

**Table 4:** Annotation of blueberry unigenes

Anno_Database	Annotated_Number	300 < =length < 1000	Length > =1000
COG_Annotation	10,263	3241	5421
GO_Annotation	21,870	8701	9422
KEGG_Annotation	7616	2829	3188
Swissprot_Annotation	2,1447	8068	9503
TrEMBL_Annotation	2,1447	8068	9503
nr_Annotation	3,1576	12,244	13,059
nt_Annotation	2,6902	9769	12,015
All_Annotation	3,4006	13,457	13,343

### GO, COG, KEGG annotation

**Fig. 2:**
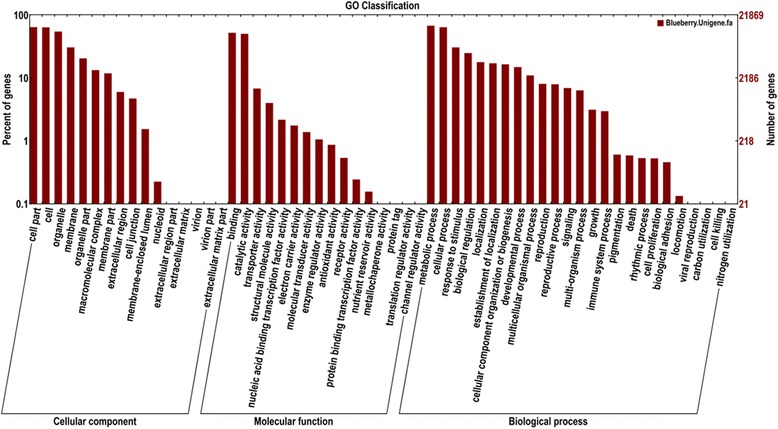
Functional annotation of assembled sequences based on gene ontology (GO) categorization. A total of 80,836 unigenes with BLAST match to known proteins were assigned to three main categories: 68,355 (49.58 %) were assigned to biological process, and 35,683 (25.89 %) and 33,840 (24.54 %) were assigned to the molecular function and the cellular component, respectively

**Fig. 3:**
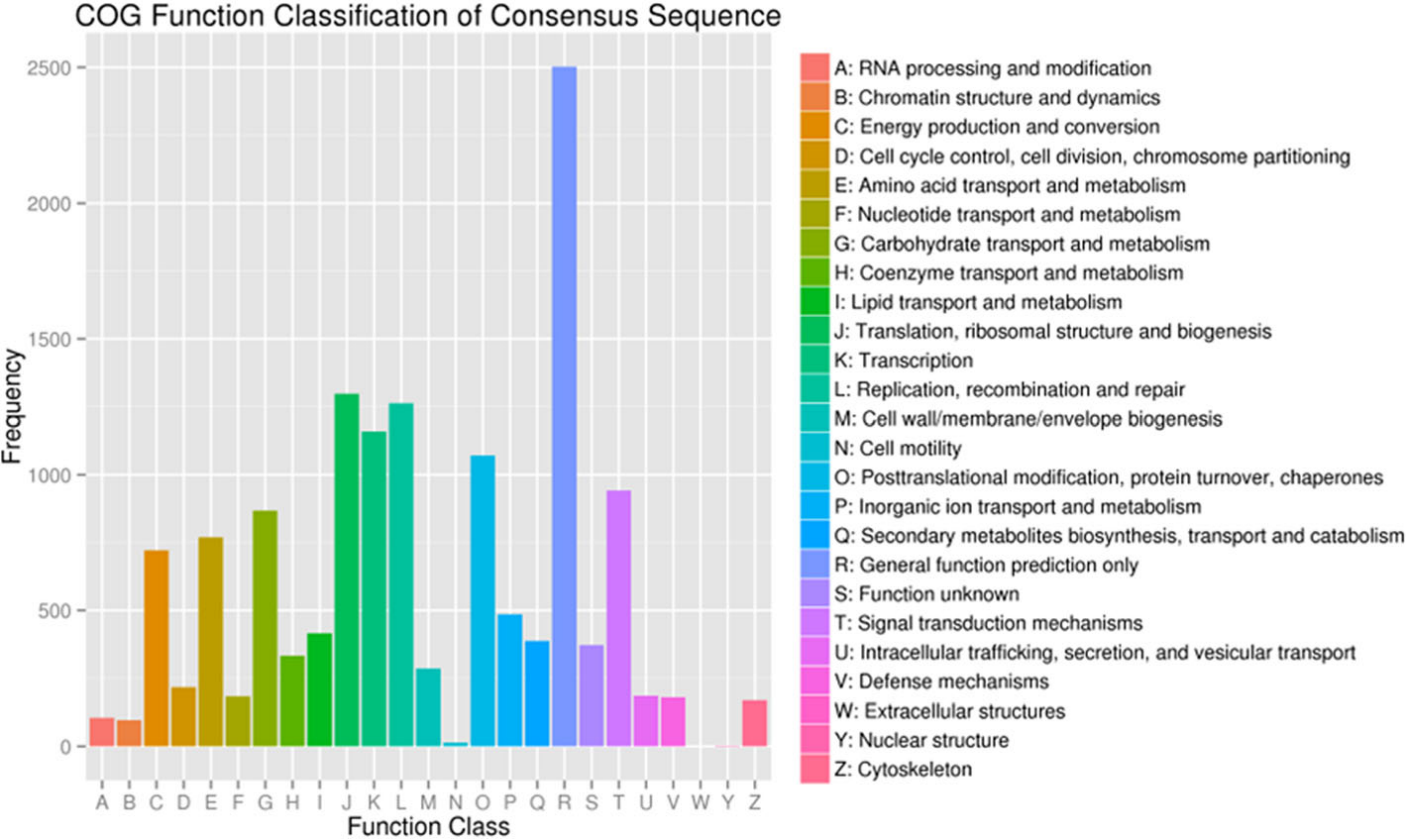
Clusters of orthologous groups (COG) classification. In total, 80,836 unigenes were assigned to the COG classification according to the Nr database and grouped into 25 COG classifications

Gene Ontology (GO) enrichment analysis was carried out to classify gene functions of the unigenes identified. The majority of the GO terms (68,355, 49.58 %) were assigned to biological process, and 35,683 (25.89 %) and 33,840 (24.54 %) were assigned to the molecular function and the cellular component, respectively (Fig. 2). It was noteworthy that cells and organelles are highly represented in category of cellular components, while binding and catalytic activity are highly typical in molecular functions category. For biological processes, genes implicated in metabolic processes and cellular processes are the most represented category followed by the response to stimulus and biological regulation (Fig. 3). These data suggest that the active synthesis of substance and energy and metabolic process are present in a wide variety of organelles and associated with fruit development and in response to stress during fruit ripening.

Furthermore, all blueberry unigenes were searched against the COG database for functional prediction and classification. Overall, 80,836 unigenes were assigned to the COG classification (Fig. 3). The largest group was the cluster for general function prediction (2502, 17.84 %), followed by translation, ribosomal structure and biogenesis (1297, 9.25 %), replication, recombination and repair (1262, 9.00 %), transcription (1160, 8.27 %), posttranslational modification, protein turnover and chaperones (1071, 7.64 %), signal transduction mechanisms (942, 6.72 %), and carbohydrate transport and metabolism (868, 6.19 %). However, small clusters were for cell motility and nuclear structure (13 and 2 unigenes, respectively). In addition, no unigene was assigned to extracellular structures.

To characterize the active biological pathways in blueberry, KEGG pathway tool was used as an alternative approach to analyze the pathway annotations of unigene sequences. The 7616 unigenes were assigned to 119 biological pathways (Additional file 2: File S2). These predicted pathways are responsible for growth and development probably via compound biosynthesis, degradation, utilization, and assimilation. These results suggest that a large number of metabolic activities are occurring during fruit development and coloring of blueberry.

### EST-SSR discovery

As highly informative markers, SSRs have developed into powerful molecular markers for comparative genetic mapping and genotyping among species within genera [19, 20]. To date, SSRs are most widely applied in genetics, evolution and breeding.

To explore EST-SSR profiles in the unigenes of blueberry, 15,457 unigene sequences were submitted to search for SSRs. A total of 6429 sequences containing 8796 SSRs were identified from 15,457 unigenes, with 1763 unigene sequences containing more than one SSR (Table 5 and Additional file 3: File S3). Di-nucleotide motifs and tri-nucleotide motifs were the most abundant with 75.11 % (4426) and 23.81 % (1403), respectively. The most abundant repeat type was AG/CT (4069), followed by AAG/CTT (388), AGG/CCT (227), and ACC/GGT (225). Because SSRs within genes are likely to be subjected to stronger selective pressure than other genomic regions, these SSRs probably represent different putative functions [21]. Therefore, the unigenes yielded from blueberry are a larger resource for SSR mining than ever and the SSR profiles which we explored are a powerful platform for research in genetics, evolution and molecular marker-assistant breeding, etc.

**Table 5:** Frequency of EST-SSRs in blueberry

Motif lenght	Repeat numbers							Total	Percent
	5	6	7	8	9	10	>10		
Di	-	1027	838	917	983	554	107	4426	75.11
Tri	867	392	131	10	2	0	1	1403	23.81
Tetra	37	8	1	0	0	0	0	46	0.78
Penta	10	2	0	0	0	0	0	12	0.20
Hexa	2	3	0	1	0	0	0	6	0.10
Total	916	1432	970	928	985	554	108	5893	
%	15.54	24.30	16.46	15.75	16.71	9.40	1.83		

### Analysis of gene regulation in anthocyanin biosynthesis pathway using the assembled unigenes

**Fig. 4:**
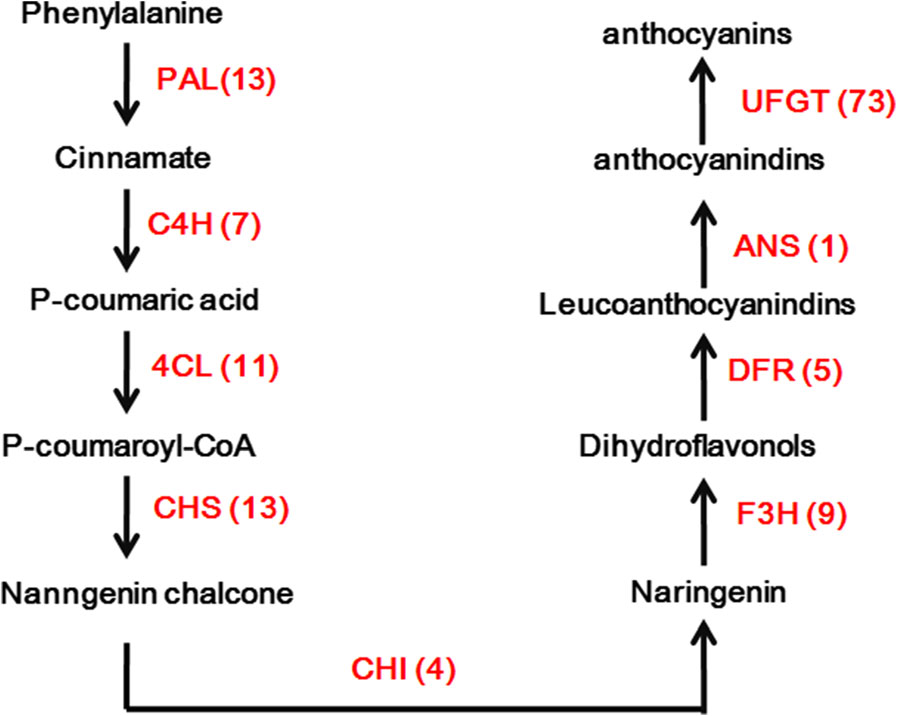
A schematic representation of anthocyanin biosynthesis pathway

In this study, we analyzed all unigene annotation in blueberry. Some genes responsible for anthocyanin biosynthesis were screened for further analysis. Anthocyanins are a large class of flavonoids which are responsible for the colors of flowers, fruits and other tissues [22]. A simple anthocyanin biosynthesis pathway is shown in Fig. 4. In most of the pathway, more than one unique sequence was annotated as encoding the same enzyme, especially the final step involving 3-glycoside formation by UFGT (UDP Glc-favonoid 3-O-glucosyl transferase) (Additional file 4: File S4).

### The relative expression levels of candidate unigenes and the content of anthocyanins during blueberry fruit and color development

**Fig. 5:**
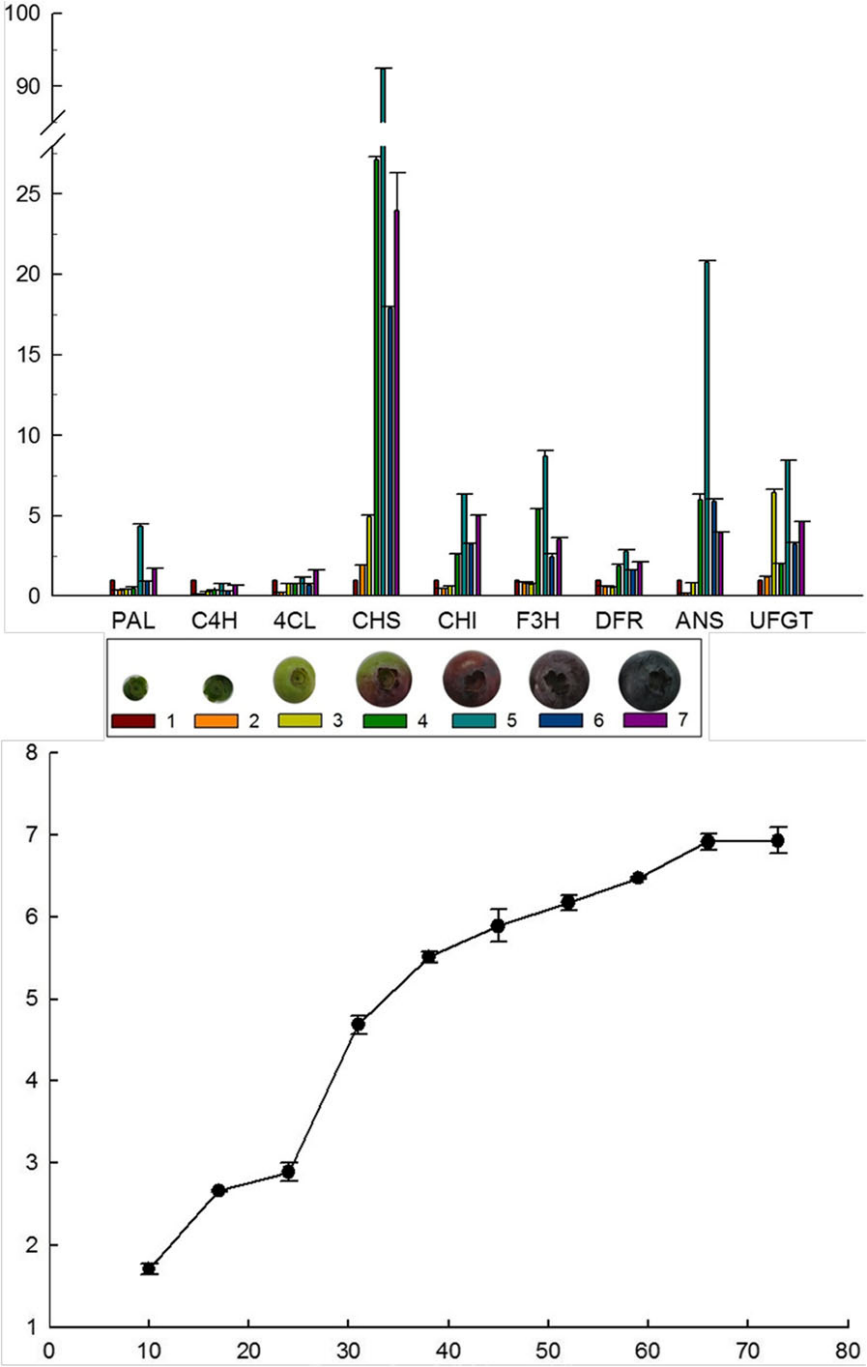
Expression levels of the color-related genes and the content of anthocyanins in blueberry. Total RNA was extracted from green, pink and blue fruits, respectively. Each value is the mean ± SE of three independent biological determinations

To validate the results from the bioinformatics analysis, the expression profile of 9 differentially expressed genes of anthocyanin biosynthesis pathway such as *PAL*, *C4H*, *4CL*, *CHS*, *CHI*, *F3H*, *DFR*, *ANS*, and *UFGT* were tested by RT-qPCR using cDNA templates from different fruit growth stages (Fig. 5). The number of unigenes assumed to be involved in anthocyanin biosynthesis pathway is shown in Additional file 4: File S4 and the specific primers used for RT-qPCR reactions are listed in Table 6. Among them, five candidate genes covered over 10 unigenes. The transcripts of some genes such as *CHS*, *CHI*, *F3H*, *DFR*, *DFR*, *ANS*, and *UFGT* increased with fruit coloring (stage 4) and reached the highest level at red fruit stage (stage 5), then slightly declined with fruit maturation (stage 5 and 6). *CHS* showed significant increase in expression at the stage of fruit coloring (stage 4), about a 19-fold increase compared with green fruit (stage 3), whereas only 3 -fold increase was observed for *ANS* at the stage of red fruit (stage 4). On the contrary, the expression level of *PAL*, *C4H*, and *4CL* changed little with fruit development. Jaakola et al. reported the expression level of CHS, F3H, DFR, and ANS, detected by northern-blotting, to increase dramatically at stage 5 (red fruit) in bilberry [16]. This is consistent with our results. Zifkin et al. characterized the expression level of DFR, ANS, ANR, and LAR in blueberry and found that the expression of DFR, ANS increased at the S6 stage (red fruit) [16].

**Table 6:** Primers used for RT-qPCR reactions

Gene	Forward Primer (5’-3’)	Reverse Primer (5’-3’)
UBC28	CCATCCACTTCCCTCCAGATTATCCAT	ACAGATTGAGAGCACCTTGGA
PAL	ATGGGTTGCCTTCAAATCTCTC	CGAGCGAGTTCACATCTTGG
C4H	TGGTAGAGTGCTTCATAATGTGC	GGTGTCGGTAGGAGGAGTTG
4CL	CTGGCGACATAGGCTACATTG	ATCTTTCATCCCGACAACTGC
CHS	TTACTAACAGCGAGCACAAGG	GAATTTCCACAACCACCATATCC
CHI	ATGGAAGGGTAAATCAGGAAAGG	TTATCATCCGCAGCCAATCG
F3H	TCACCTATTTCTCATACCCACTTC	TTTCCAAACCCATTGCCTCAG
DFR	ACTGGAGCGACTTGGATTTTG	TGGGATGTASGGCATAATGAAGG
ANS	CGTCTGCTTGGGATTGGAAG	TGTGGAGGATGAAGGTGAGG
UFGT	TGAAGAACTAGACCCCGAACTC	CCAAAGGCGATATAGGCAACG

The anthocyanin content was next quantified at different developmental stages to test if anthocyanins accumulate in accordance with the pattern of gene expression. As expected, at green berry stage, anthocyanins were detected at low levels, only 0.5 mg/g FW. In accordance with the deep coloration, in mature fruits (blue fruit) the levels of anthocyanin peaked dramatically. Analysis of expression and anthocyanin accumulation indicated that there was a significant correlation between the expression profile of candidate genes and the accumulation of anthocyanins.

## Conclusions

In the present study, the transcriptome and gene expression profiling over fruit development and coloring of blueberry were initiated by means of Illumina sequencing. A total of approximately 13.67 Gbp of data were obtained and assembled into 186,962 transcripts and 80,836 unigenes from three stages of blueberry fruit. A large number of simple sequence repeats (SSRs) and candidate genes were identified. A total of 6429 sequences containing 8796 SSRs were identified from 15,457 unigenes, and 1763 unigenes containing more than one SSR. Compared with the previously published data, our dataset has a higher amount of data (Nucleotide), better data quality (Q20 or Q30%), and better assembly results (Average length and N50). Therefore, our high quality reads produced in this study are an important advancement and a new resource for interpretation of high-throughput data for blueberry species regarding from sequencing data depth or species extension.

## Methods

### Plant materials and RNA extraction

Blueberry (*Vaccinium corymbosum* ‘Sierra’) fruits were harvested from an organic blueberry farm (Tianshuo Farm in Hebei Province, China) during the 2013 to 2015 growing season. The tissues at three different developmental stages including green fruit (G, 30d), pink fruit (P, 50d) and blue fruit (B, 75d), were randomly sampled in the field from 4- or 5-year-old healthy blueberry plants. The plants had been propagated by tissue culture, thus all came from the same mother plant. All samples intended for RNA extraction were flash-frozen in liquid nitrogen immediately after collection and stored at −80 °C until use. Fifty fruits were collected at each time point and combined for RNA extraction. RNA was extracted using the Plant total RNA Kit (TIANGEN, Beijing, China). The purified RNA quality and quantity was evaluated using a spectrophotometer (Thermo Scientific, Waltham, MA, USA) and an Agilent 2100 Bioanalyzer (Agilent Technologies, Santa Clara, CA, USA).

### mRNA-seq library construction for Illumina sequencing

The mRNA-seq library was constructed following the Illumina’s mRNA-seq Sample Preparation Kit (Illumina Inc., San Diego, CA, USA) from three stages of fruit development, separately. Briefly, the mRNA was purified from total RNA samples using oligo (dT) magnetic beads, and then broken into small pieces by divalent cation under an elevated temperature. Taking these RNA fragments as templates, the first strand cDNA was synthesized by reverse transcriptase and random primers, while the second strand cDNA was synthesized using DNA Polymerase I and RNase H. The cDNA fragments were blunt-ended and ligated to sequencing adaptors. The fragments (200 ± 25 bp) were then separated by agarose gel electrophoresis and selected for PCR amplification. Finally, the mRNA-seq libraries were sequenced by the Illumina HiSeq™ 2000 sequencing.

### Sequence data analysis and assembly

The high-quality clean read data for assembly was separated from adapters and low-quality. Reads with more than 10 % Q30 bases were removed. De novo assembly of transcriptome was done using the Trinity method with an optimized k-mer length of 25 [18]. The contigs were clustered and further assembled according to paired-end information. The longest transcripts in the cluster units were defined as unigenes. EMBOSS Getorf Software was used to predict coding regions (http://emboss.bioinformatics.nl/cgi-bin/emboss/getorf).

### Sequence annotation

The assembled sequences were searched against the public protein databases, such as the NCBI Nr and Nt databases (National Center for Biotechnology Information (NCBI) nonredundant protein (Nr) database, non-redundant nucleotide sequence (Nt) database), Swissprot, KEGG (Kyoto Encyclopedia of Genes and Genomes, http://www.genome.jp/kegg/kegg2.html), COG, (Cluster of Orthologous Groups of proteins) and TrEMBL using BLASTX with an E-value ≤ 10^−5^. Gene ontologies (GO) were assigned to each unigene using Blast2GO (Conesa et al., 2005, http://wego.genomics.org.cn/cgi-bin/wego/index.pl).

### EST-SSR detection

The EST-SSRs were detected among the 15,457 blueberry unigenes which were longer than 1 kb using the Simple Sequence Repeat Identification Tool (SSRIT, http://www.gramene.org/db/markers/ssrtool). The parameters were adjusted to identify perfect di-, tri-, tetra-, penta- and hexa- nucleotide motifs with a minimum of six, five, four, four and four repeats, respectively [23, 24].

### Gene validation and expression analysis by quantitative RT-qPCR

The expression of potential candidate genes of anthocyanin biosynthesis pathway in blueberry such as *PAL*, *C4H*, *4CL*, *CHS*, *CHI*, *F3H*, *DFR*, *ANS* and *UFGT* were examined by RT-qPCR. Specific primers used for RT-qPCR reactions were listed in Table 6 according to the open reading frames of the target genes. Real-time qPCR reactions were carried out in a StepOnePlus Real-time PCR system (ABI, USA) using SuperRealPreMix (SYBR Green) kit (TIANGEN BIOTECH, Beijing, China) according to the manufacturer’s instructions. *UBC28* gene was used as an internal control for normalization, and each sample was assayed in triplicate. Relative transcript levels were calculated and normalized as described previously [25].

### Extraction and assay of anthocyanin

The pH differential method was used [26] with minor modification. The fruits at different growth stages were harvested from multiple healthy plants and fully ground in liquid nitrogen. Briefly, aliquots of 1.0 g ground fruit tissue was dissolved in 20 mL of 60 % methanol (pH 3.0), and then kept at 40 °C for 2 h. The extracts was collected through vacuum pump filter followed by decompression concentration in the rotary evaporation apparatus. Next, aliquots of 1 ml of extract was taken in duplicate and diluted to 25 ml in buffer A (0.2 mol.L^−1^KCL:0.2 mol.L^−1^ HCl = 25:67,v:v,pH1.0) and buffer B (1 mol.L^−1^ NaAc:1 mol.L^−1^ HCL:H2O = 100:60:90,v:v,pH4.5), respectively. The absorbance value at 520 nm was measured via UV-600 ultraviolet and visible spectrophotometers.

The content of anthocyanins was calculated using the formula. The formula is C (mg/g) = (A0 ‐ A1) × V × N × M/(e × m). Among them, A0 and A1 was the light absorption value of anthocyanins at pH 1.0 and pH 4.5, respectively. V means the total volume of the extract, N is the dilution ratio, M is 449 for the standard molecular weight marker anthocyanins, e is 29,600 for standard extinction coefficient, m is 1 g of the sample weight. The assays were repeated three times along with three independent repetitions of the biological experiments and the means of the three biological experiments were calculated for the total anthocyanin levels in each of the samples.

## Additional files

Additional file 1:
**File S1.** Summary of the sequence assembly for five cultivars of blueberry. (XLSX 12 kb)
Additional file 2:
**File S2.** Pathway assignment based on KEGG. (XLSX 11 kb)
Additional file 3:
**File S3.** Repeats of EST-SSRs in blueberry. (XLSX 18 kb)
Additional file 4:
**File S4.** List of unigenes that cover 9 selected genes respectively. (XLSX 12 kb)

## Abbreviations

4CL: 4-coumarate CoA ligase
ANS: Anthocyanidin synthase
C4H: Cinnamate 4-hydroxylase
CHI: Chalcone isomerase
CHS: Chalcone synthase
COG: Clusters of orthologous groups of protein
DFR: Dihydroflavonol 4-reductase
F3H: Flavonoid 3-hydroxylase
GO: Gene ontology
KEGG: Kyoto encyclopedia of genes and genomes
Nr: Non-redundant protein database
Nt: Non-redundant nucleotide database
PAL: Phenylalanine ammonialyase
qRT-PCR: Quantitative real-time PCR
RPKM: Reads per kb per million reads
Swiss-Prot: Annotated protein sequence database
TrEMBL: Computer-annotated supplement to Swiss-Prot database
UFGT: UDP-Glucose flavonoid 3-O-glucosyl transferase
